# Gene expression signature of atypical breast hyperplasia and regulation by SFRP1

**DOI:** 10.1186/s13058-019-1157-5

**Published:** 2019-06-27

**Authors:** Kelly J. Gregory, Amy L. Roberts, Erin M. Conlon, Jacob A. Mayfield, Mary J. Hagen, Giovanna M. Crisi, Brooke A. Bentley, Jeffrey J. Kane, Grace Makari-Judson, Holly S. Mason, Jun Yu, Lihua Julie Zhu, Karl Simin, Jacob P. S. Johnson, Ashraf Khan, Ben R. Schneider, Sallie S. Schneider, D. Joseph Jerry

**Affiliations:** 1 Pioneer Valley Life Sciences Institute, Springfield, MA 01199 USA; 2 Department of Veterinary and Animal Sciences, University of Massachusetts-Amherst, Amherst, MA 01003 USA; 3 Department of Mathematics and Statistics, University of Massachusetts, Amherst, MA 01003 USA; 4 Department of Pathology, University of Massachusetts Medical School/Baystate, Springfield, MA 01199 USA; 5 Division of Hematology-Oncology, University of Massachusetts Medical School/Baystate, Springfield, MA 01107 USA; 6 Department of Surgery, University of Massachusetts Medical School/Baystate, Springfield, MA 01199 USA; 7 University of Massachusetts Medical School, Molecular, Cell, and Cancer Biology, Worcester, MA 01605 USA; 8 Department of Pathology, University of Massachusetts Medical School, Worcester, MA 01605 USA; 9 Division of Rheumatology, Immunology and Allergy, Brigham and Women’s Hospital, Harvard Medical School, Boston, MA USA

**Keywords:** Atypical hyperplasia, Breast, Lobular, Ductal, Premalignancy, SFRP1, Gene expression profile

## Abstract

**Background:**

Atypical breast hyperplasias (AH) have a 10-year risk of progression to invasive cancer estimated at 4–7%, with the overall risk of developing breast cancer increased by ~ 4-fold. AH lesions are estrogen receptor alpha positive (ERα+) and represent risk indicators and/or precursor lesions to low grade ERα+ tumors. Therefore, molecular profiles of AH lesions offer insights into the earliest changes in the breast epithelium, rendering it susceptible to oncogenic transformation.

**Methods:**

In this study, women were selected who were diagnosed with ductal or lobular AH, but no breast cancer prior to or within the 2-year follow-up. Paired AH and histologically normal benign (HNB) tissues from patients were microdissected. RNA was isolated, amplified linearly, labeled, and hybridized to whole transcriptome microarrays to determine gene expression profiles. Genes that were differentially expressed between AH and HNB were identified using a paired analysis. Gene expression signatures distinguishing AH and HNB were defined using AGNES and PAM methods. Regulation of gene networks was investigated using breast epithelial cell lines, explant cultures of normal breast tissue and mouse tissues.

**Results:**

A 99-gene signature discriminated the histologically normal and AH tissues in 81% of the cases. Network analysis identified coordinated alterations in signaling through ERα, epidermal growth factor receptors, and androgen receptor which were associated with the development of both lobular and ductal AH. Decreased expression of *SFRP1* was also consistently lower in AH. Knockdown of *SFRP1* in 76N-Tert cells resulted altered expression of 13 genes similarly to that observed in AH. An SFRP1-regulated network was also observed in tissues from mice lacking *Sfrp1*. Re-expression of *SFRP1* in MCF7 cells provided further support for the SFRP1-regulated network. Treatment of breast explant cultures with rSFRP1 dampened estrogen-induced progesterone receptor levels.

**Conclusions:**

The alterations in gene expression were observed in both ductal and lobular AH suggesting shared underlying mechanisms predisposing to AH. Loss of SFRP1 expression is a significant regulator of AH transcriptional profiles driving previously unidentified changes affecting responses to estrogen and possibly other pathways. The gene signature and pathways provide insights into alterations contributing to AH breast lesions.

**Electronic supplementary material:**

The online version of this article (10.1186/s13058-019-1157-5) contains supplementary material, which is available to authorized users.

## Background

Studies of premalignant breast lesions provide insights into the mechanisms rendering the breast epithelium susceptible to oncogenic transformation as well as identify interventions that could prevent breast cancer. Atypical hyperplasias (AH) develop in the terminal duct lobular units of the breast and are further subdivided into either atypical ductal hyperplasia (ADH) or lobular neoplasia (LN). Lobular neoplasia encompasses atypical lobular hyperplasia (ALH) and lobular carcinoma in situ (LCIS). In addition, flat epithelia atypia (FEA) is a subtype of atypical ductal epithelium lacking architectural changes as seen in ADH. The 10-year risk of progression to invasive cancer is estimated to be 7% for all AH [1] with a cumulative incidence approaching 35% at 30 years. The overall risk of developing breast cancer is increased by ~ 4-fold among women with atypia and is similar for both ductal and lobular lesions [1, 2]. However, the risk is most prominent among women with higher breast density [3] suggesting that mechanisms underlying breast density affect the progression of premalignant breast lesions. AH lesions are most often positive for estrogen receptor alpha (ERα+) and approximately 90% of tumors that develop subsequently are ERα-positive. Thus, AH represent a precursor lesion to low-grade ERα-positive tumors. Selective receptor modulators or aromatase inhibitors prevent progression of AH to invasive carcinomas by about 60% [4–6], further supporting an important role for estrogen signaling in malignant progression of AH.

The expression of several genes and proteins have been evaluated in AH and their relationship with risk of progression. CK5/6 and ERα can aid the morphologic interpretation of usual ductal hyperplasia and AH lesions and increase the sensitivity of distinguishing among these lesions [7, 8]. However, levels of ERα in AH detected by immunohistochemical staining did not predict risk of breast cancer [9]. The extent of Ki67 immunoreactivity in normal breast tissue has been associated with an increased risk of breast cancer [10]. Combined measures of proliferation, tumor suppressor activity, and inflammatory signaling within AH, using immunohistochemical scoring for Ki67, p16, and COX-2 respectively, have been used to evaluate breast cancer risk [11]. Elevated levels of EZH2 were shown to be an early marker for progression of preneoplastic lesions [12], while other studies identified increases in DNA methylation within promoter elements of tumor suppressor genes such as *APC*, *DLEC1*, *HOXA1*, *RASSF1A*, and *SFRP1* [13, 14]. Progressive methylation of genes in early lesions was reported for *RASSF1A* and *RARB2*, suggesting the potential value of these targets [15]. Higher levels of estrogen receptor beta (ERβ) are associated with a decreased risk of progression of AH [16], suggesting that selective agonists of ERβ may offer potential therapy for chemoprevention. Gene expression profiles have also been used to identify early changes in AH as well as adjacent tumors [17, 18]. These studies suggest the presence of molecular changes within breast epithelial cells associated with the transition to AH and risk of progression to breast cancer.

In the present study, patients diagnosed with AH and no history of breast cancer (prior to or within the 2-year follow-up after AH diagnosis) were selected. Laser capture microdissection was used to collect both histologically normal benign epithelium (HNB) and AH tissues from each patient. The complete transcriptome was evaluated by microarray, and gene expression patterns were used to define signatures that distinguish AH lesions from HNB tissues. Although ADH and LN tissues have distinct morphologic features, they did not form distinct clusters based on gene expression patterns suggesting that these premalignant lesions share underlying alterations in transcriptional programs. Pathway analyses identified genes encoding ERα, epidermal growth factor receptors (ERB-B), and androgen receptor (AR) as central nodes in the expression profiles. ERB-B2 and WNT signaling pathways were also strongly over-represented among the genes differentially expressed in AH. As methylation and loss of SFRP1 expression had been associated with premalignancy, we determined if it may be responsible for altered expression of a subset of genes altered in AH. Knock down of *SFRP1* expression in normal breast epithelial cells (76N-Tert) identified 13 genes within the AH signature that had not previously been connected to SFRP1. SFRP1-regulated genes were also observed in mammary tissue from mice bearing deletion of the *Sfrp1* gene. Re-expression of SFRP1 in an ERα-positive breast cancer cell line (MCF7), which has lost expression of the endogenous *SFRP1* gene, had the opposite effect providing additional confirmation of an SFRP1-regulated gene network. Antagonism of estrogen-induced responses in progesterone receptor levels was demonstrated by addition of recombinant SFRP1 to human breast explant cultures. These findings demonstrate that SFRP1 expression is diminished in AH resulting in deregulation of a larger program of genes and loss of restraint on ERα signaling which may contribute to development of premalignant breast lesions.

## Methods

### Patient specimens

This is a retrospective study using formalin-fixed and paraffin-embedded (FFPE) archival tissue blocks. A search of pathology electronic files (CoPath) included patients with isolated AH lesions (atypical ductal hyperplasia, flat epithelial atypia, atypical lobular hyperpalsai, classic type lobular carcinoma in-situ) on core biopsy with subsequent excisional biopsy, isolated AH lesions on primary excisional biopsies, and reduction mammoplasties. Exclusion criteria included patients with a prior history of breast cancer or breast cancer within 2 years of initial AH diagnosis or insufficient AH lesion on subsequent excision. Original diagnoses were supported by at least two pathologists. A subspecialized breast pathologist (GMC) reviewed all cases retrieved for the study for concordance of original diagnosis. Cases that, upon review by GMC, did not meet histopathologic criteria for AH (ductal or lobular) were excluded. Characteristics of patients and diagnoses are in Table 1. Patient 14 was found to have a diagnosis of severe ADH bordering on ductal carcinoma in situ (low grade) upon review of original slide material. Institutional Review Board approval was obtained from Baystate Health, Springfield, MA (protocol number 182463).

**Table 1:** Patient characteristics and array identifiers

Patient ID	Age at diagnosis	Diagnosis	Affected breast	Atypia array ID	Benign array ID
1	43	FEA	Left	JJ013	JJ014
2	46	LCIS	Right	JJ001	JJ002
3	47	ADH	Right	JJ005	JJ006
4	58	LCIS	Left	JJ015	JJ016
5	40	ADH, FEA	Right	JJ003	JJ004
6	63	ADH	Left	JJ007	JJ008
7	52	LCIS	Right	JJ009	JJ010
8	62	ADH, FEA	Left	JJ012	JJ011
9	46	LCIS	Left	JJ017	JJ018
10	40	ADH	Right	JJ019	NA^@^
11	49	ADH, ALH	Left	DJJ021	DJJ022
12	53	ALH, FEA	Right	DJJ023	DJJ024
13	53	LCIS	Left	DJJ025	DJJ026
14*	58	ADH	Right	DJJ027	DJJ028
15	60	LCIS, ALH	Left	DJJ029	DJJ030
16-Block1**	52	ADH, FEA	Right	DJJ031	DJJ032
16-Block2**	52	ADH, FEA	Right	DJJ033	NA***
18	47	ALH	Left	DJJ035	DJJ036
19	55	ALH	Right	DJJ037	DJJ038
20	70	ADH severe	Right	DJJ039	DJJ040
21	44	FEA	Right	DJJ041	DJJ042
22	50	ADH/FEA	Left	DJJ043	DJJ044
23	54	ADH/DCIS	Left	DJJ045	DJJ046

*Confirming records revealed that patient 14 followed up with DCIS in left breast 6 months later

**For patient 16, two independent blocks with AH were analyzed separately

***There was insufficient benign tissue for Patient 16-Block2

^@^Defective array

### Microscopic evaluation

Atypical hyperplasias (AH) arise in the terminal duct lobular units of the breast and are divided into ductal and lobular subtypes based on cytomorphologic characteristics. Ductal lesions included in the study are atypical ductal hyperplasia (ADH) and flat epithelial atypia (FEA); lobular lesions included atypical lobular hyperplasia (ALH) and/or classic lobular carcinoma in situ (LCIS), representing a spectrum and also known as lobular neoplasia. Subjects with either ductal or lobular atypia were included in the analysis to assess differences in transcriptional features. Areas of AH and benign ducts/lobules were marked for microdissection by the breast pathologist. RNA of sufficient quantity and quality was obtained from 21 AH lesions (from 20 patients). The tissues included 8 lobular lesions (ALH and/or LCIS), 11 ductal lesions (ADH and/or FEA), and 2 that were a mixture of ductal and lobular lesions. All subjects were female; the mean age was 51.9 years (SD = 7.9 years).

### Analysis of RNA integrity

The integrity of RNA in tissue sections was assessed by amplifying 5′ fragment (nucleotides 1355–1472) and 3′ fragment (nucleotides 1650–1717) of the β-actin gene (Additional file 2: Table S5) by quantitative RT-PCR (RT-qPCR). An 8-μm section from each tissue block was placed on a glass microscope slide in RNase-free conditions, deparaffinized in 3 changes of xylene, and allowed to air-dry under vacuum in a desiccator for 30 min. The tissue sample was scraped from the slide using a razor blade directly into 150 μl digestion buffer containing 10 μl proteinase K (miRNeasy FFPE Kit, Qiagen, Germantown, MD) and incubated at 55 °C for 2 h. The samples were subsequently incubated at 80 °C for 15 min and transferred to ice for 3 min. The samples were centrifuged at 13,000×*g* for 20 min, and the supernatant was transferred to new tubes. The RNA was harvested following DNase digestion using the miRNAeasy FFPE kit as described in the manufacturer’s instructions (Qiagen). The cDNA was prepared using 100 ng total RNA, oligo dT primers, and the Transcriptor first strand cDNA synthesis kit according to the manufacturer’s instructions (Roche, Indianapolis, IN). Amplification of 5′ and 3′ β-Actin targets were performed using the KAPA SYBR Fast Master Mix (Thermo Fisher, Waltham, MA) containing 200 nM forward primer, 200 nM reverse primer, and 5 μl cDNA. The conditions for mRNA amplification were performed as follows: 40 cycles each of 1 cycle at 95 °C for 2 min, 1 cycle at 95 °C for 15 s, and 1 cycle at 60 °C for 30 s; 1 cycle at 95 °C for 15 s, 1 cycle at 60 °C for 15 s, 20 min ramp, and 1 cycle at 95 °C for 15 s. The *C*_t_ value of the 3′ β-Actin target was subtracted from the *C*_t_ value of the 5′ β-Actin target to determine the amplification ratio. Specimens with ratios < 5 were used for microdissection and transcriptome-wide profiling.

### Microdissection and RNA isolation

The H&E stained sections of the AH samples were used to estimate the total area for microdissection. A minimum area of 10 × 10^6^ μm^2^ was required to ensure a minimum of 50 ng total RNA. Consecutive tissue sections (8-μm thick) were cut using RNase-free conditions and mounted on membrane slides (MMI, Rockledge, FL). The first and every 4th section were H&E stained for microscopic evaluation to confirm that AH tissue was present in unstained microdissected areas. The AH lesion and benign glandular tissue were marked by a breast pathologist (GMC) for microdissection. The benign glandular areas were selected to be at least 1 cm away from the AH lesion in the same tissue block or a different block. The tissues on membrane slides were deparaffinized in 3 changes of xylenes and allowed to air-dry under a vacuum in a desiccator for 30 min prior to laser capture microdissection. The unstained sections were oriented for microdissection aided by landmarks defined on the H&E stained slides. Areas to be microdissected were circumscribed using MMI Cell Tools software (Version Celltools-4.4 #261, Rockledge FL). Microdissected AH and HNB tissues from each patient were collected separately onto caps (MMI Inc., Rockledge FL). The microdissected tissue was collected in 150 μl digestion buffer containing 10 μl proteinase K (miRNeasey FFPE Kit, Qiagen), was kept overnight at 55 °C, and was stored at − 80 °C until further processing. Total RNA was isolated from microdissected tissue using the miRNeasy FFPE Kit (Qiagen) according to the manufacturer’s instructions and quantified using a NanoDrop™1000 (Thermo Fisher Scientific).

### cDNA synthesis, amplification, and labeling

The Ovation® FFPE WTA System (NuGEN, San Carlos, CA) was used to prepare amplified cDNA from FFPE-derived total RNA because amplification is initiated at the 3′ end as well as randomly throughout the whole transcriptome in the sample which makes this system ideal for amplification of RNA obtained from FFPE samples. Fifty nanograms of RNA was used to prepare the cDNA according to the manufacturer’s instructions. The cDNA was then purified using columns from the QIAquick PCR Purification kit (Qiagen). Buffer PB from the purification kit was added to the cDNA reaction, loaded on to the column, and centrifuged for 1 min at 13,000×*g*. The flow through was discarded, and 80% ethanol was added to the column and centrifuged for 1 min at 13,000×*g*. The 80% ethanol wash step was repeated, and the purified cDNA was eluted with nuclease-free water. An aliquot containing 5 μg of cDNA was fragmented and labeled using the Encore® Biotin Module (NuGEN) according to the manufacturer’s instructions. The biotin-labeled cDNA was hybridized to Affymetrix 1.0 ST microarrays by Genome Explorations (Memphis, TN).

### Analysis of microarray data

The data were normalized using the Single-Channel Array Normalization (SCAN) and Universal exPression Codes (UPC) methods from the BioConductor R package “SCAN.UPC” [19]. This package produces standardized expression measures that are used to estimate whether a given gene or probe is active in a specific sample [20, 21]. ComBAT, an empirical Bayesian framework, was used to adjust data for batch effects [22]. The normalized data are available from the NCBI Gene Expression Omnibus Repository [23] series record GSE118432. Limma [24] was used to identify differentially expressed genes in a paired-sample model, with HNB and AH samples paired by patient. AH arrays JJ019 and DJJ033 were excluded from the analysis because paired HNB data were not available for these patients. A total of 1039 probesets were selected with adjusted *p* < 0.05. Two methods were employed to identify gene signatures distinguishing AH and normal benign tissue. Agglomerative clustering was performed using AGNES [25] to visualize gene expression patterns. Prediction Analysis of Microarrays (PAM) was used as an alternative approach to define a minimal gene expression signature [26].

### Network analysis

The differentially expressed probesets were mapped to 812 unique genes and used for network analysis [27]. In cases where there were more than one probeset for a gene, the data were averaged. Protein interaction networks were constructed using the STRING database available within the network analyst tool (http://www.networkanalyst.ca/). Over-representation of KEGG pathways was determined, and pathways were visualized using Cytoscape [28].

### Analysis of genes dependent on SFRP1

The 76N-Tert cell line was derived from normal breast epithelial cells [29] and expresses endogenous *SFRP1*. Generation and cultivation of engineered human cell lines (TERT-pSUPER, TERT-siSFRP1, MCF7-pCDNA, MCF7-SFRP1) has been described previously [30–32]. Total RNA was extracted from cell lines using an acid-phenol extraction procedure [33], according to the manufacturer’s instructions (Trizol, Invitrogen, Carlsbad, CA). Relative levels of mRNA were determined by using the 1-Step Brilliant® SYBRIII® Green RT-qPCR Master Mix Kit (Stratagene) containing 200 nM forward primer, 200 nM reverse primer, and 100 ng total RNA. The conditions for cDNA synthesis and target mRNA amplification were performed as follows: 1 cycle of 50 °C for 30 min, 1 cycle of 95 °C for 10 min, and 35 cycles each of 95 °C for 30 s, 55 °C for 1 min, and 72 °C for 30 s. Expression of each gene was normalized to levels of β-actin mRNA. The PCR primer sequences used are described in Additional file 2: Table S5.

### Animals

The study was carried out in strict accordance with the recommendations in the Guide for the Care and Use of Laboratory Animals of the National Institutes of Health. The protocol was approved by the Baystate Medical Center Institutional Animal Care and Use Committee (Permit Number: 132681). Ten-week-old female C57BL/6-*Sfrp1*+/+ mice (*n* = 6) and C57BL/6-*Sfrp1*−/− mice (*n* = 6) were individually housed in plastic cages with food and water provided continuously and maintained on a 12:12 light cycle. The *Sfrp1* knockout allele has been described previously [34, 35]. Mammary tissue was collected from mice, flash-frozen, and stored at − 80 °C until processed for RNA isolation and used to quantify relative levels of transcripts by RT-qPCR using primers described in Additional file 2: Table S5.

### Human breast explant cultures

The tissue was aseptically minced and placed on Surgifoam gelatin sponges (Ferrosan, Sueborg, Denmark) in 60-mm tissue culture dishes containing phenol-red free DMEM/F12 (Gibco) 2% charcoal stripped serum, insulin, and gentamycin treated with vehicle (100% EtOH), 10 nM 17β-estradiol (E_2_; Sigma), or 10 nM E_2_ with 1 μg/mL rSFRP. Explant cultures were maintained for 24 h in 5% CO_2_ air and subsequently formalin-fixed and paraffin-embedded.

### Progesterone receptor staining

Immunohistochemistry (IHC) was performed on a DakoCytomation autostainer using the Envision HRP Detection system (Dako, Carpinteria, CA). Mammary tissue blocks were sectioned at 4 μm, deparaffinized in xylene, rehydrated in graded ethanols, and rinsed in Tris-phosphate-buffered saline (TBS). Heat-induced antigen retrieval was performed in a microwave at 98 °C in 0.01 M citrate buffer. After cooling for 20 min, sections were rinsed in TBS and incubated with rabbit polyclonal anti-PR 1:500, (Cell Signaling; #8757) for 30 min at room temperature. Immunoreactivity was visualized by incubation with diaminobenzidine for 5 min. Tissue sections were counterstained with hematoxylin, dehydrated through graded ethanols and xylene, and cover-slipped. Images were captured with an Olympus BX41 light microscope using (SPOT™Imaging Solutions, Detroit, MI). PR staining of epithelial cells was quantified using ImageJ.

### Statistical analyses

The mean expression of genes in parental cells (TERT-pSUPER, MCF7-SFRP1) versus *SFRP1* knockdown/overexpressing cells (TERT-siSFRP1, MCF7-SFRP1) and *Sfrp1*^*+/+*^ versus *Sfrp1*^−/−^mammary gland tissues were compared using paired *t* tests.

## Results

### Expression patterns of biomarkers

**Fig. 1:**
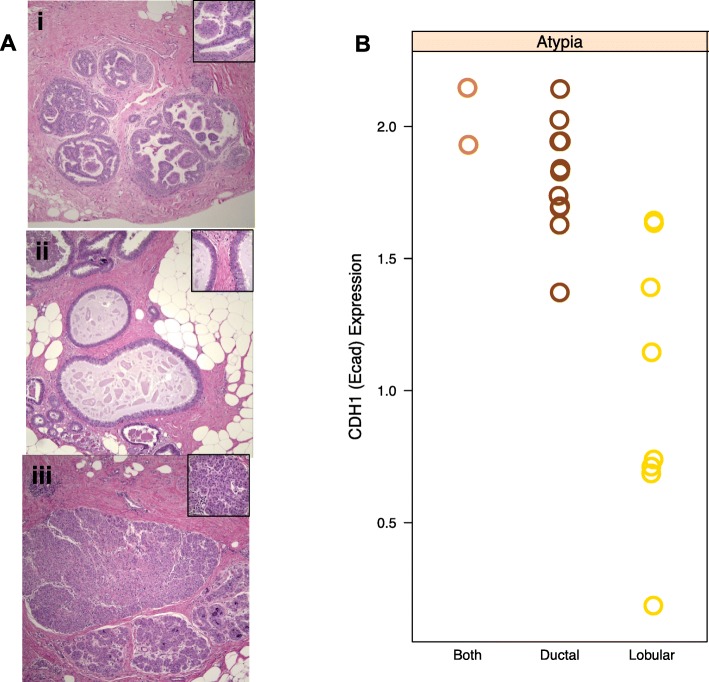
Differential expression of *CDH1* in AH and LN. **a** Examples of H&E stained sections of ductal and lobular lesions that were microdissected and used for transcriptional profiling: (i) atypical ductal hyperplasia, (ii) flat epithelial atypia, and (iii) lobular carcinoma in situ. The magnification for the main images are × 100 and × 600 for the insert. **b** Expression of *CDH1* is shown for atypical hyperplasias that were diagnosed as ductal, lobular, or contained components of both. The lobular atypical hyperplasias had overall lower levels of *CDH1* expression compared to the ductal atypical hyperplasias

AH lesions were classified as ductal or lobular based on histomorphologic features (Fig. 1a). Immunohistochemical membranous expression of E-cadherin (encoded by *CDH1*) has been used to differentiate ductal and lobular hyperplasias, with a reduced level observed in lobular lesions [36]. Consistent with these observations, ductal lesions had overall higher levels of *CDH1* mRNA compared to the lobular lesions (Fig. 1b). AH tissues from two patients had both lobular and ductal characteristics and expressed levels of *CDH1* mRNA similar to that in ductal lesions. These results demonstrated that the differential expression of *CDH1* in lobular and ductal AH was preserved in the linear amplification and detection methods.

**Fig. 2:**
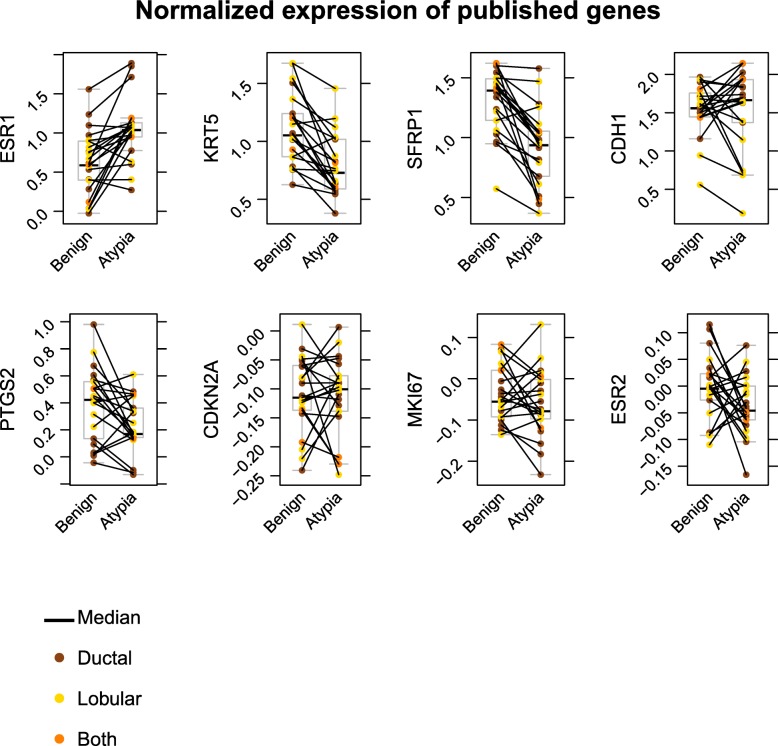
Normalized expression of genes implicated as potential biomarkers of AH. The relative levels of expression are reported for genes associated with atypical hyperplasias. Only *ESR1*, *KRT5*, and *SFRP1* had expression levels that differed significantly between histological normal benign tissues and atypical hyperplasias. The colors indicate the diagnosis of lesions as ductal, lobular, or containing both

In an effort to define gene targets distinguishing AH from HNB tissues, ComBAT and LIMMA-adjusted mRNA expression levels were evaluated for several potential biomarkers (Fig. 2). Levels of mRNA for *ESR1* (encoding ERα) were increased in AH while *KRT5* (encoding cytokeratin 5) was decreased (Fig. 2). *SFRP1* (encoding secreted frizzled-related protein 1) was among the most strongly downregulated genes in AH (Fig. 2). These results validate selected genes that have been shown to be differentially expressed in AH breast lesions reaffirming the utility of the microarray expression profiling methods. Expression of mRNA for COX2, P16/INK4A, and KI67 (encoded by *PTGS2*, *CDKN2A*, *MKI67*, respectively) and estrogen receptor beta (ERβ, encoded by *ESR2*) were analyzed because prior studies suggested these as biomarkers of AH at greater risk of progression to breast cancer. We found that mRNA levels for these genes did not differ significantly between AH and HNB in either lobular or ductal subtypes of AH.

### Expression signature for atypical hyperplasias

**Fig. 3:**
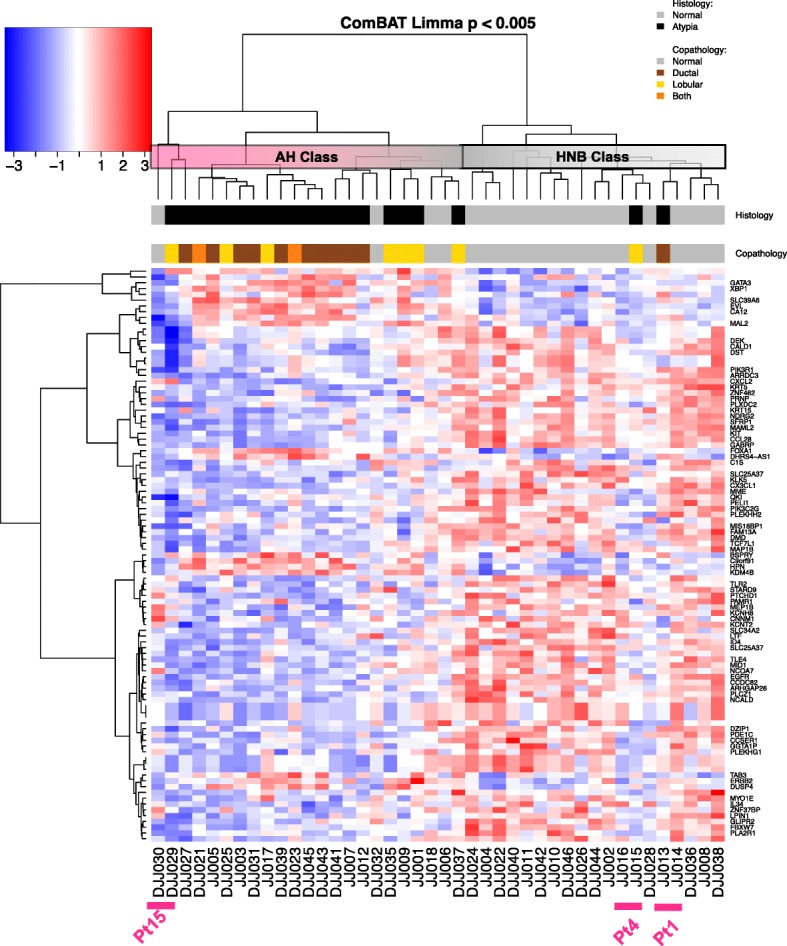
Hierarchical clustering of differentially expressed genes. Genes that were differentially expressed at *p* < 0.005 were selected and clustered using AGNES. Two main clusters were defined that were enriched for either histologically normal benign tissue (HNB class) or histologically AH (AH class). This gene set did not separate the ductal and lobular subtypes of AH. Two AH samples had expression patterns similar to the histologically normal tissues. Samples JJ13 and JJ15 were diagnosed as FEA and LCIS, respectively. Both clustered adjacent to the histologically normal tissue from the same patient (JJ14 and JJ16, respectively) suggesting that these are true benign tissues. In contrast, DJJ030 is histologically normal tissue, but clusters adjacent to the LCIS tissue (DJJ029) from patient #15 suggesting that the tissue harbors genetic alterations driving the gene expression but has not yet acquired the histological architecture of AH. The data values are expressed as log base 2 ratios of HNB/AH with red indicating increased expression compared to the overall mean levels across tissues and blue decreased levels

Gene expression profiles were used to derive a signature of AH and to identify additional diagnostic biomarkers. Gene expression patterns in normal breast epithelium are quite variable among individuals which can obscure the modest transcriptional alterations in premalignant tissues. Therefore, a paired analysis of HNB and AH within individuals was used to detect genes that are differentially expressed in AH tissues. The paired analysis of AH and HNB tissues identified a total of 1039 differentially expressed probesets (LIMMA with adjusted *p* values of < 0.05; Additional file 2: Table S1). By increasing the threshold (adjusted *p* value < 0.005), the signature was reduced to 99 probesets (Additional file 2: Table S2) which were used for hierarchical clustering. The data values are expressed as log2 ratios of HNB/AH with red indicating higher expression compared to the overall mean levels across tissues and blue decreased expression. Examples are *GATA3*, *XBP1*, and *EVL* for which mRNA levels are increased in most of the AH tissues. In contrast, *ARRDC3*, *CXCL2*, *MAML2*, and *SFRP1* are expressed in HNB, but expression is significantly reduced in AH. Two major clusters were detected that are enriched for AH or HNB patterns of gene expression (Fig. 3, designated “AH class” and “HNB class”). The overall pattern observed was a decrease in expression of genes in AH compared to the HNB class. Given the substantial divergence in histologic features of lobular and ductal lesions, it was anticipated that these would form sub-branches. While sub-branches are evident in the AH class, lobular and ductal lesions are distributed similarly in these branches suggesting that ductal and lobular lesions share a set of alterations driving their development.

While genes are selected to distinguish AH, misclassification was anticipated due to limitations in the specificity of the gene signature and variations in molecular features underlying AH lesions. The clustering identifies 2 patients for which the AH samples cluster with the HNB. The lesion in patient 1 (array JJ013) was an FEA. This is an intermediate lesion which may involve a single-cell layer and bears some overlap in histological features with normal tissue. Furthermore, the HNB tissue from patient 1 (JJ014) is adjacent to the FEA tissue in the cluster. The HNB tissue in patient 4 (JJ016) is adjacent to the clustering of the LCIS lesion (JJ015) suggesting that the molecular features are largely benign despite the morphologic features. Conversely, the HNB tissue for patient 15 clustered with the AH tissues (DJJ030, DJJ029, respectively). The HNB and AH tissues from patient 15 are adjacent to one another in the clustering suggesting that the HNB tissue shared underlying molecular alterations despite the differences in histologic features. The two samples from patient 15 form a branch in the cluster with the ADH from patient 14 (DJJ027). Patient 14 was diagnosed with DCIS in the contralateral breast 6 months later raising the possibility that the pattern of expression in this branch represents increased likelihood for progression. In each of these patients (1, 4, 15), the AH is adjacent to the HNB in the cluster providing support for the reproducibility gene expression patterns within individual patients. Overall, the set of genes distinguishes HNB tissues for 81% of patients (Fig. 3; 17/21 in “HNB class”), providing biomarkers that can be used to aid discrimination of AH.

**Fig. 4:**
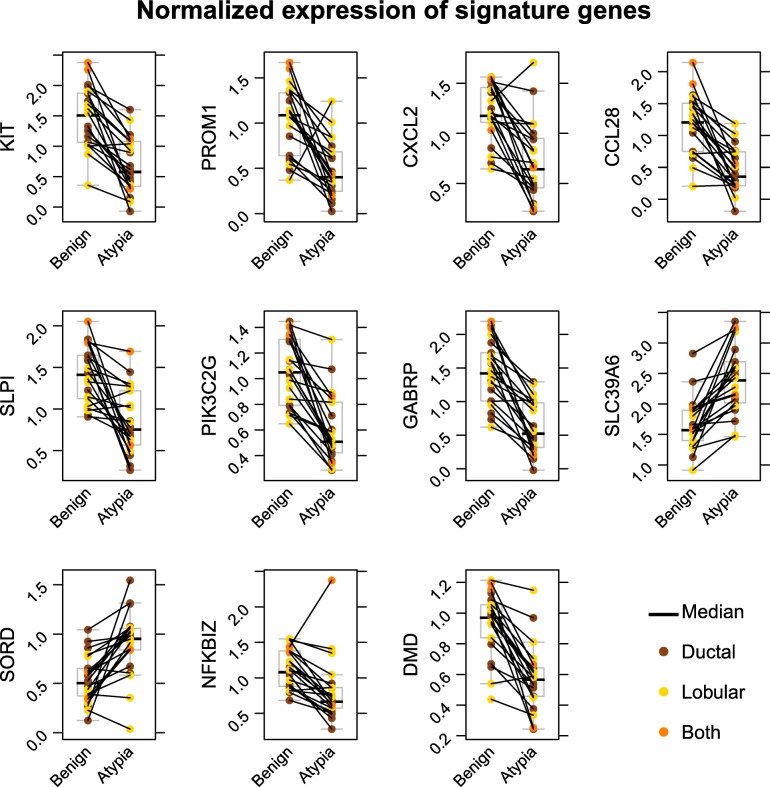
Differential expression of genes in histologically normal benign (HNB) and AH tissues. AGNES was used to select 99 genes that are most strongly associated with AH. The distribution of normalized expression is shown for each of the top 11 genes. The colors indicate the diagnosis of lesions as ductal, lobular, or containing both

The data were also analyzed using the paired model with blocking for the histopathology of lesions (ductal vs lobular) to identify potential biomarkers. This identified 11 genes that differed consistently in mRNA levels between HNB and AH (Fig. 4). Both *KIT* and *PROM1* (encoding CD133) have been associated with stem cell phenotypes and exhibit decreased expression in AH. The chemokines *CXCL2* and *CCL28* along with secreted leukocyte peptidase inhibitor (*SLPI*) participate in inflammatory responses and were among the differentially expressed genes. While inflammation contributes to oncogenic progression, both were downregulated in atypia. Similarly, the PI-3-kinase *PIK3C2G* was consistently decreased in AH along with genes involved in ion transport (*GABRP*, *SLC39A6*). The increase in sorbitol dehydrogenase (*SORD*) suggests metabolic changes, but the levels were variable making it unlikely to be a reliable biomarker. The decreased levels of *NKBIZ* (encoding an inhibitor of NFkB) together with increases in *ESR1* may combine to increase sensitivity to estrogen-stimulated proliferation in AH.

Prediction Analysis of Microarrays (PAM) was used as an alternate algorithm to identify gene expression signatures. The ComBat-normalized data from the LIMMA paired model (*p* < 0.05) was analyzed and identified a PAM signature of 139 probesets (Additional file 2: Table S3). The intersection of the AGNES and PAM signatures identified 43 genes common to both methods (Table 2). The keratins (*KRT5* and *KRT15*) are among this group as well as luminal markers (*KIT*, *FOXA1*). *SFRP1* was also identified in the signature of both prediction algorithms. These independent methods for class prediction provide a reduced set of biomarkers to aid diagnosis of AH.

**Table 2:** Differentially expressed genes identified in both AGNES and PAM signatures

AFFYMETRIX_EXON_ID	Gene name
7903414	Amylase, alpha 1A (salivary) (AMY1A)
7927058	Unknown
7951133	Mastermind-like transcriptional coactivator 2 (MAML2)
7954208	Phosphatidylinositol-4-phosphate 3-kinase catalytic subunit type 2 gamma (PIK3C2G)
7963427	Keratin 5 (KRT5)
7976726	Enah/Vasp-like (EVL)
7977621	NDRG family member 2 (NDRG2)
7978706	Forkhead box A1 (FOXA1)
7989501	Carbonic anhydrase 12 (CA12)
7996027	C-X3-C motif chemokine ligand 1 (CX3CL1)
8015337	Keratin 15 (KRT15)
8022927	Solute carrier family 39 member 6 (SLC39A6)
8024909	Lysine demethylase 4B (KDM4B)
8041644	Pleckstrin homology, MyTH4 and FERM domain containing H2 (PLEKHH2)
8075182	X-box binding protein 1 (XBP1)
8086607	Lactotransferrin (LTF)
8094441	Solute carrier family 34 member 2 (SLC34A2)
8095110	KIT proto-oncogene receptor tyrosine kinase (KIT)
8100994	C-X-C motif chemokine ligand 2 (CXCL2)
8106098	Microtubule-associated protein 1B (MAP1B)
8108873	Rho GTPase activating protein 26 (ARHGAP26)
8109926	Gamma-aminobutyric acid type A receptor pi subunit (GABRP)
8111932	C-C motif chemokine ligand 28 (CCL28)
8113073	Arrestin domain containing 3 (ARRDC3)
8117120	Inhibitor of DNA binding 4, HLH protein (ID4)
8119076	Unknown
8121861	Nuclear receptor coactivator 7 (NCOA7)
8124144	DEK proto-oncogene (DEK)
8127234	Dystonin (DST)
8130556	Uncharacterized LOC100129518 (LOC100129518)
8136347	Caldesmon 1 (CALD1)
8145291	Solute carrier family 25 member 37 (SLC25A37)
8150076	Dual specificity phosphatase 4 (DUSP4)
8150428	Secreted frizzled-related protein 1 (SFRP1)
8152119	Neurocalcin delta (NCALD)
8155359	Contactin-associated protein-like 3B (CNTNAP3B)
8155460	Contactin-associated protein-like 3 pseudogene 2 (CNTNAP3P2)
8155540	Contactin-associated protein-like 3B (CNTNAP3B)
8161460	Contactin-associated protein-like 3B (CNTNAP3B)
8166447	Patched domain containing 1 (PTCHD1)
8168727	Unknown
8171297	Midline 1 (MID1)
8171921	Dystrophin (DMD)

### Pathway analysis

**Fig. 5:**
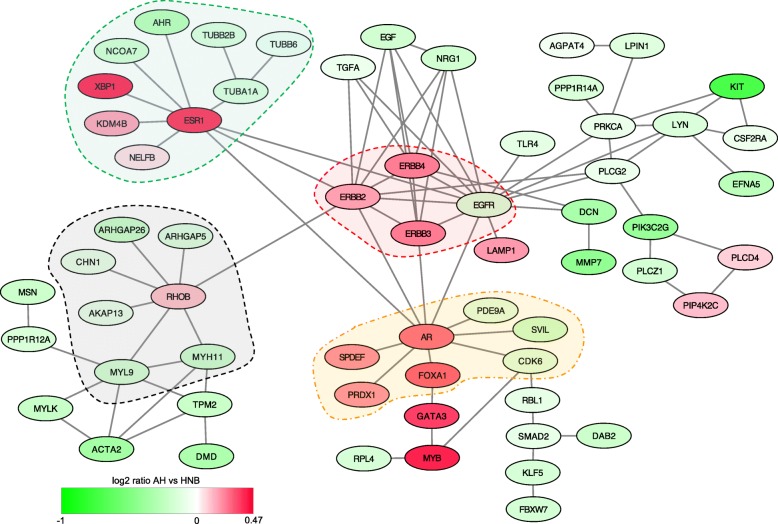
Zero-order network formed by genes that are differentially expressed in histologically normal benign (HNB) and AH tissues. A network of protein interactions was constructed using 812 genes that were differentially expressed between AH and HNB tissues. The *ERB-B* genes (*ERBB2*, *ERBB3*, *ERBB4*, *EGFR*) define a central node connected to three major nodes associated with elevated expression of *ESR1*, *AR*, and *ROHB* in AH compared to HNB tissues. The data are presented as ratios with red indicating increased expression in AH tissues compared to HNB. Dotted lines encompassing major nodes indicate zero-order subnetworks

Gene expression patterns were overlaid onto protein-protein interaction databases to detect pathways that are altered in AH. Probesets that were differentially expressed between the AH and HNB tissues (representing 812 genes) formed a zero-order network of 61 differentially expressed genes (Fig. 5) that had direct interactions (61 seeds, 90 edges). The zero-order network reveals extensive interactions of genes with *ESR1*, *RHOB*, *AR*, and EGFR receptors (*ERBB2*, *ERBB3*, *ERBB4*) which form central nodes. Expression of ERB-B receptors (*ERBB2*, *ERBB3*, *ERBB4*) are elevated and have a total of 23 edges. In contrast, expression of ligands (*TGFA*, *EGF*, *NRG1*) and *EGFR* are decreased. *RHOB* levels were also elevated in AH lesions and form a separate node with a group of genes that are all downregulated. Increased expression of *ESR1* is consistent with prior studies; however, the associated increase in expression of *KDM4B*, *XBP1*, and *NELB* suggests a subnetwork that may act in concert with ERα. The androgen receptor (*AR*) is also elevated in AH lesions and forms a subnetwork. Although *FOXA1* and *GATA3* are shown as interactors with only *AR* based on the STRINGS database, both genes are known to collaborate with ERα.

The 1st-order networks were also analyzed to identify broader pathways that may be over-represented and indicate broader alterations in signaling pathways. The major network had 306 genes/seeds that were differentially expressed generating 3849 edges and 2364 nodes. Additional subnetworks were detected but had only 1 differentially expressed seed in each. Within the major network with 306 seeds, there was over-representation of five KEGG pathways with *p* < 10^−25^ (Table 3). The alterations in ERBB2 and WNT signaling overlap extensively with the pathways in cancer.

**Table 3:** Pathways over-represented in the first-order 812 gene network

Pathway	Total	Expected	Hits	*p* value	FDR
Pathways in cancer	310	80.9	197	6.53E−47	1.42E−44
ErbB signaling pathway	87	22.7	70	1.35E−26	1.47E−24
Chronic myeloid leukemia	73	19.1	62	3.38E−26	2.45E−24
HTLV-I infection	199	52	121	5.30E−26	2.78E−24
WNT signaling pathway	144	37.6	97	6.41E−26	2.78E−24
Focal adhesion	200	52.2	117	4.23E−23	1.53E−21
Pancreatic cancer	69	18	56	8.10E−22	2.51E−20
Neurotrophin signaling pathway	123	32.1	82	1.39E−21	3.78E−20
Prostate cancer	87	22.7	64	1.27E−20	3.07E−19
Colorectal cancer	49	12.8	43	1.21E−19	2.63E−18
Osteoclast differentiation	119	31.1	77	3.84E−19	7.58E−18
Acute myeloid leukemia	57	14.9	47	4.98E−19	9.01E−18
Hepatitis C	100	26.1	68	8.76E−19	1.46E−17
T cell receptor signaling pathway	98	25.6	67	1.02E−18	1.58E−17
Melanogenesis	101	26.4	68	2.02E−18	2.92E−17
Regulation of actin cytoskeleton	182	47.5	102	2.39E−18	3.24E−17
Chagas disease (American trypanosomiasis)	89	23.2	62	4.82E−18	6.15E−17
MAPK signaling pathway	265	69.2	132	1.51E−17	1.82E−16
B cell receptor signaling pathway	75	19.6	54	7.04E−17	8.04E−16
Gap junction	89	23.2	60	1.99E−16	2.16E−15
Renal cell carcinoma	60	15.7	46	2.51E−16	2.60E−15
Epstein-Barr virus infection	91	23.8	60	9.93E−16	9.79E−15
Cell cycle	124	32.4	74	1.37E−15	1.30E−14
Axon guidance	118	30.8	71	2.90E−15	2.62E−14
Toll-like receptor signaling pathway	97	25.3	62	3.02E−15	2.62E−14
Glioma	65	17	47	5.85E−15	4.88E−14
Herpes simplex infection	103	26.9	64	7.95E−15	6.39E−14

### Genes in the signature regulated by SFRP1 expression

SFRP1 is best known for its antagonism of the WNT pathway. However, it binds other proteins, such as RANKL and thrombospondin, and its loss has been demonstrated to affect signaling of other critical pathways involving ERα, TGFB receptor, and p53 through less well-studied mechanisms. Therefore, loss of expression in AH may directly influence genes differentially expressed in AH.

**Fig. 6:**
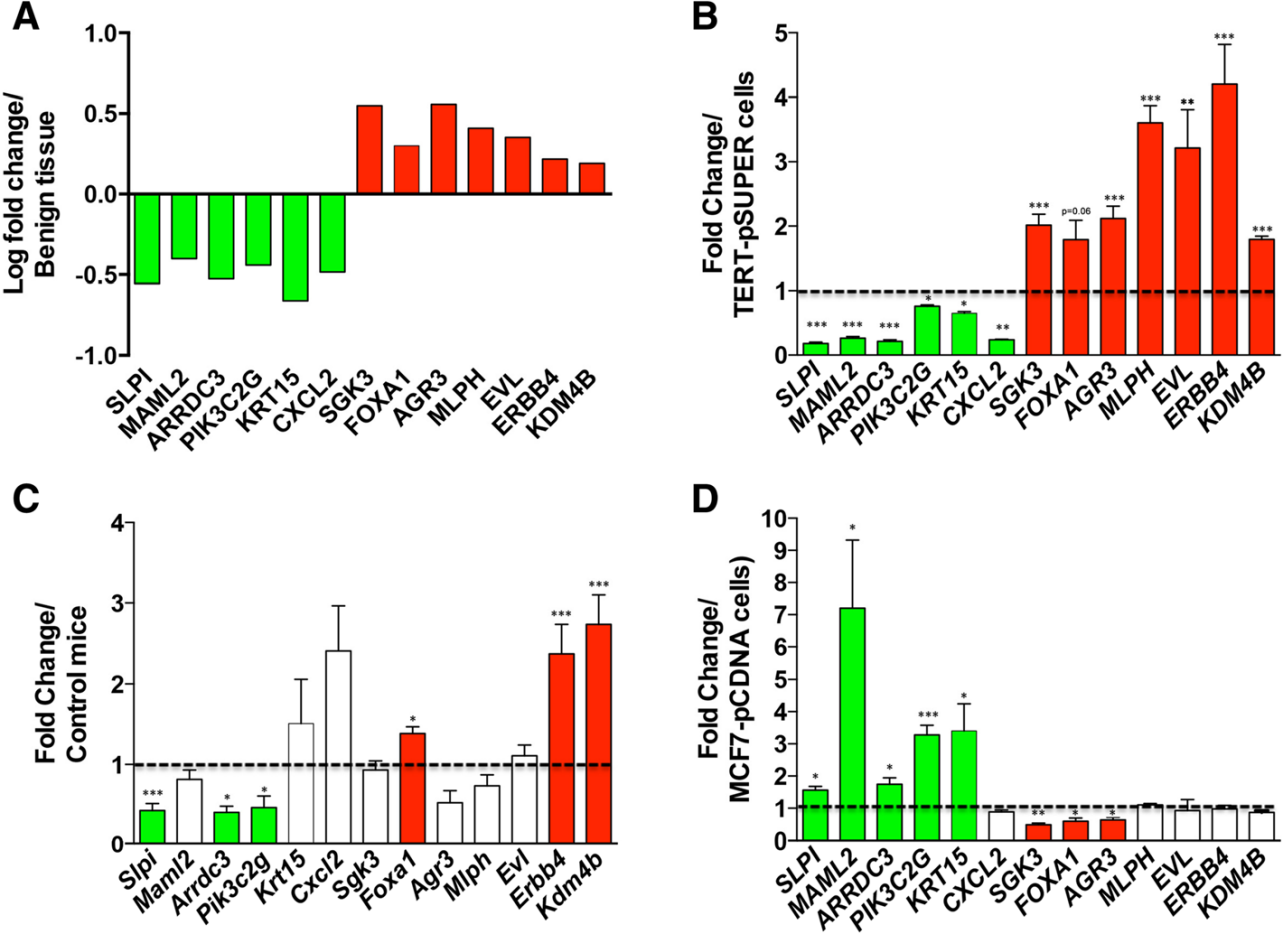
Expression of genes in AH and regulation by *SFRP1*. Genes associated with AH were identified by AGNES and PAM were examined for regulation by *SFRP1*. **a** The relative expression of the genes in AH are shown. Green indicates genes with decreased expression in AH relative to the HNB tissues and red indicates those with increased expression. **b** The effect of *SFPR1* knockdown on gene expression was analyzed in a cell line derived from normal breast epithelium and immortalized with telomerase (76NTERT cells). Relative levels of transcripts were determined by RT-qPCR in TERT-siSFRP1 cells and the TERT-pSUPER vector control cells. **c** Similarly, mRNA levels of the genes was compared by RT-qPCR in mouse mammary glands derived from *Sfrp*1^−/−^ and control *Sfrp1*^+/+^ mice. **d** Breast cancer cells overexpressing SFRP1 (MCF7-SFRP1) and control cells (MCF7-pCDNA) were also compared for relative levels of gene expression by RT-qPCR. The level of *SFRP1* mRNA was normalized to the amplification of *ACTB* mRNA, which was performed in parallel wells for each cell line or tissue. Bars represent mean ± SEM *SFRP1/ACTB* and are expressed as relative expression of control TERT-pSUPER (**b**), control mice (**c**), and MCF7-pCDNA (**d**). Genes where loss of *SFRP1* results in decreased expression are shown in green, those increased are shown in red, and those which are unchanged are shown in white *p < 0.05, **p < 0.01, and ***p < 0.001 (significantly different from control using Student’s *t* test)

The 76N-Tert cells express *SFRP1* and, therefore, were used to test whether knockdown of *SFRP1* (TERT-siSFRP1) may drive a set of genes that are differentially expressed in AH. The levels of *SFRP1* in TERT-siSFRP1 cells are shown in Additional file 1: Figure S1. A total of 31 genes within the PAM signature (Additional file 2: Table S3) were selected to test for differential expression in the TERT-siSFRP1 vs TERT-pSUPER cells. In total, 6 genes with decreased expression in AH tissues (*SLPI*, *MAML2*, *ARRDC3*, *PIK3C2G*, *KRT15*, *CXCL2*) were also decreased by knockdown of *SFRP1* (TERT-siSFRP1 cells; Fig. 6a, b). Conversely, 6 genes for which mRNA levels were increased in AH tissues (*SGK3*, *FOXA1*, *AGR3*, *MLPH*, *EVL*, *KDM4B*) also had increased expression in TERT-siSFRP1 cells compared to the TERT-pSUPER control cells (Fig. 6b). Among 19 other genes from the PAM signature, mRNA levels for 16 genes were unaffected by knockdown of *SFRP1* while expression was the opposite of that observed in AH for 3 genes. *ERBB4* was also tested because it is part of the zero-order network (Fig. 5) and had been implicated in regulation by *SFRP1*. Knockdown of *SFRP1* resulted in increased *ERBB4* mRNA consistent with the higher levels in AH compared to HNB (Fig. 6a, b). These results suggest that decreased expression of *SFRP1* alters a network of genes in AH.

We examined the expression of the 13 genes in mammary tissues from *Sfrp1*−/− mice to confirm dependence on SFRP1 and determine if the network is conserved across species (Fig. 6c). Similar to AH in humans (Fig. 6a), loss of *Sfrp1* in mice resulted in reduced expression of 3 genes compared to the wild-type mice (*Slpi*, *Arrdc3*, *Pik3c2g*) and increased expression of 3 genes (*Foxa1*, *Erbb4*, *Kdm4b*). Therefore, a portion of the SFRP1-regulated network was conserved in this mouse model. To explore if re-expression of SFRP1 could reverse the changes, we used MCF7 breast cancer cells which lack *SFRP1* expression and compared effects of over-expression (MCF7-SFRP1 vs MCF7-pCDNA; see Additional file 1: Figure S1). Constitutive expression of *SFRP1* increased 5 genes (*SLPI*, *MAML2*, *ARRDC3*, *PI3KC2G*, *KRT15*) that were downregulated in AH and TERT-siSFRP1 cells and reduced expression of 3 genes (*SGK3*, *FOXA1*, *AGR3*) that were upregulated in AH and TERT-siSFRP1 cells (Fig. 6d). These data demonstrate the presence of an SFRP1-regulated gene network in both human tissues and cell lines that is also conserved in mice.

**Fig. 7:**
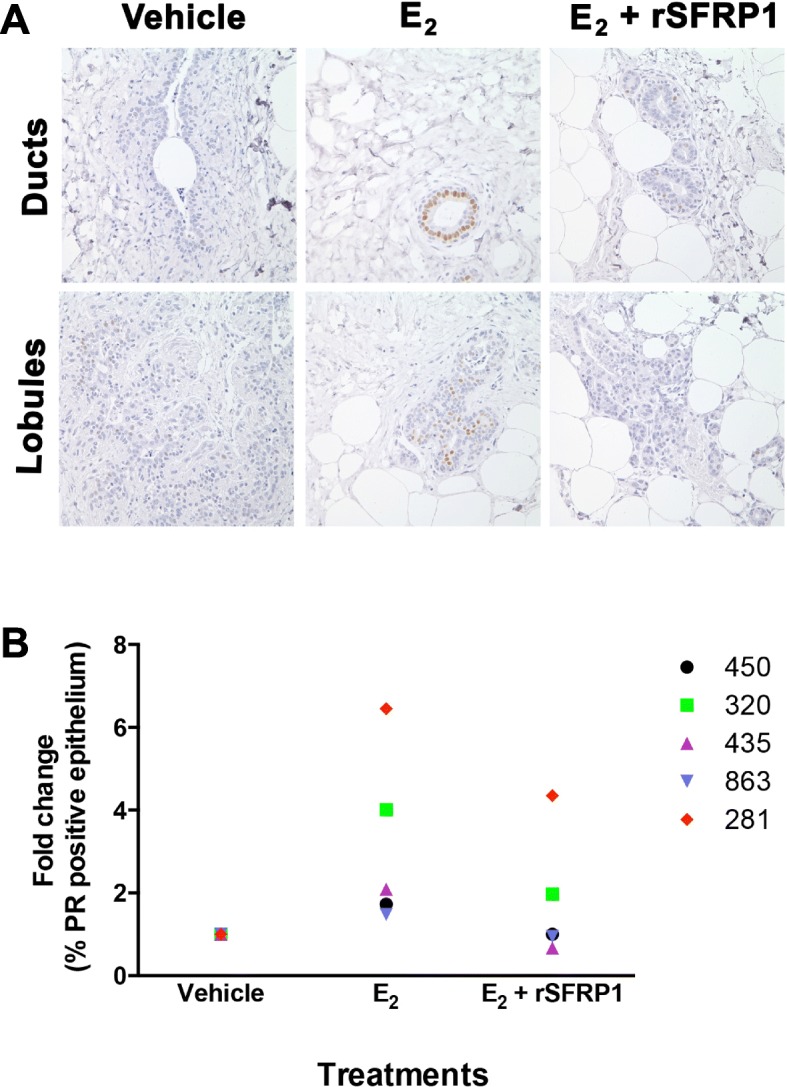
Effect of SFRP1 on estrogen-induced expression of progesterone receptor (PR) in breast explant cultures. Normal breast tissues from women undergoing reduction mammoplasty were placed in culture and treated with vehicle, 17β-estradiol (E_2_) or E_2_ together with rSFRP1 (E2 + SFRP1). Immunohistochemical staining was used to detect cells expressing PR (brown chromogen). (**a**) Representative images from Subject 435 were captured at 400 × images for staining in ducts and lobule for one patient. (**b**) PR-stained cells were counted in each treatment group for 5 different subjects and the fold change in PR-positive cells is shown for each patient. Responses to E_2_ varied among individuals but SFRP1 diminished the effect of E_2_ in each case (E_2_ vs E_2_ +rSFRP1, *p* <0.01)

Enhanced estrogen signaling and loss of *SFRP1* expression are common features within the gene expression signature in AH, but it is unclear if these are related mechanistically or are simply complementary alterations acquired during development of AH. Therefore, we wanted to determine if SFRP1 protein levels control responsiveness to estrogen stimulation. Explant cultures of normal breast tissue from 5 subjects were treated with 17β-estradiol (E_2_) in the presence of recombinant SFRP1 (rSFRP) protein or vehicle control for 24 h to determine if expression of progesterone receptor (PR) was altered (Fig. 7). The percentage of breast epithelial cells expressing PR was increased in response to E_2_ treatment in the 5 patients. Addition of rSFRP1 suppressed the response to E_2_ (*p* = 0.01). These results demonstrate that SFRP can influence E_2_-stimulated responses in normal breast tissues and that its loss in AH may affect estrogen signaling.

## Discussion

The pathways disrupted in AH offer insights into the early molecular changes that are associated with increased risk of malignancy. Prior analyses of AH have most often utilized regions of AH that co-exist with carcinomas [17, 18]. This approach was necessary because tissues from percutaneous core biopsies and excisional biopsies with AH in women without cancer are often exhausted for histopathological diagnosis. The recovery and quality of RNA from formalin-fixed, paraffin-embedded (FFPE) tissues is poor and limits the methods for genome-wide transcriptional profiling to 3′ ends of mRNA for prior studies [37]. However, AH adjacent to tumors may already harbor alterations similar to the tumor cells [38] which can confound results. To overcome these limitations, we optimized methods for RNA isolation and amplification to allow reproducible analysis of transcriptional profiles using microdissected AH lesions from women without a prior history of breast cancer.

Using these methods, we confirmed increases in mRNA levels of *ESR1* and decreases in *KRT5* as were previously reported using immunohistochemical detection of these proteins [7], as well as differences in *CDH1* (encoding E-cadherin) in ductal and lobular AH [36]. Paired analysis of AH and HNB tissues within individuals identified a 99-gene signature that discriminated 90% of the AH and 81% of the HNB (Fig. 3). HNB from 4 individuals clustered with the “AH class” which may reflect false-positives. Alternatively, it may also reflect a limitation of using tissue that is adjacent to AH lesions which can harbor genetic alterations present in the AH [39, 40]. Field effects have been reported at margins of 2 cm [41]. This is especially likely for patient 15 for whom the HNB clustered next to the AH suggesting similarity at the molecular level. Conversely, AH from patients 1 and 4 were in the HNB class which may indicate false negatives, but may also indicate lesions that express the morphologic features of AH yet have a molecular signature more similar to HNB. The lesion from patient 1 was an FEA which is consistent with a low potential for malignant progression. These inherent uncertainties in diagnosis lead to an underestimate of the transcriptional changes in AH. Conversely, the differences in expression detected using the 99-gene signature reflect a robust set of biomarkers that can aid concordance in the diagnosis of AH [42, 43].

Within the signature, there were increases in genes associated with the luminal (e.g., *ESR1*, *GATA3*, *KRT18*) and decreases in genes associated with the basal breast epithelium (e.g., *KRT5*, *TP63*, *ACTA2*). This may reflect a clonal expansion neoplastic luminal cells in AH which could result in a decrease in the apparent expression of genes that are markers of the basal epithelium. However, there were also significant decreases in genes associated with the normal luminal epithelium. Immunolocalization of c-KIT is reported in normal breast epithelial cells [44, 45] with loss of c-KIT in low-grade breast cancers. ELF5 has been associated with differentiation of the luminal epithelium, and its expression is decreased significantly in luminal A, luminal B, and HER2 subtypes of breast cancer [46]. Levels of these genes associated with luminal epithelium were decreased significantly in the AH samples (Fig. 4; Additional file 2: Table S3). In addition, increased levels of immunohistochemical staining for ERα, GATA3, and FOXA1 proteins have been reported in early breast lesions [8, 37, 47] which is consistent with increased expression of mRNA detected in AH (Figs. 2, 3, and 4, Additional file 2: Table S3). Therefore, the signature is not solely due to the increase in luminal epithelium in AH tissues. The expression analyses of AH may represent more complex cellular phenotypes that are not characteristic of either the basal or luminal cells within normal breast tissues. Transcriptional profiling of mouse mammary and human breast tissues have also revealed an unexpected complexity of cellular identities and lineages [48–50]. Therefore, it is possible that the gene signature in AH may represent an enrichment of a subclass of breast epithelial cells.

Models of breast progression generally support the evolution of invasive ductal and lobular carcinomas along two distinct lineages of lesions [51]. This is based on the differences in genomic alterations and gene expression profiles in invasive ductal and lobular carcinomas [52, 53]. A comprehensive analysis identified mutational hallmarks distinguishing invasive lobular carcinomas [54]. However, we failed to observe a distinction in expression profiles from ductal and lobular AH by hierarchical clustering (Fig. 3). Similar numbers of AH were profiled for both the ductal and lobular histological classes, and expression of E-cadherin (*CDH1*) mRNA levels confirmed the classifications. This raises the possibility that alterations in a common set of pathways contribute to atypical hyperplasias in both ductal and lobular epithelial cells. The subsequent alterations observed during progression toward ductal and lobular carcinomas may be defined by vulnerabilities that differ in the ductal and lobular cell types.

The network analyses provide further insights into the spectrum of molecular changes detected in AH and render the breast epithelium at heightened risk of breast cancer. Among genes that are differentially expressed in AH (0-order network), we observed a network involving *ESR1*, *ERB-B* receptors and *AR/GATA3/FOXA1* (Fig. 5). Prior studies examining transcriptional profiles of benign hyperplasias also identified increased expression of *ESR1* and *ERBB* genes [8, 47]. A study of matched normal, early neoplasia and carcinoma from a cohort of 25 women identified elevated expression of *ERBB2*, *FOXA1*, and *GATA3* [37]. This study by Brunner et al. included only 7 cases of early neoplasias without synchronous cancer which likely limits the threshold for detecting changes in AH. Nonetheless, they observed elevated expression of *KDM4B*, *XBP1*, *AR*, *MYB*, and *SPDEF* in early neoplasias compared to normal [37] which were also elevated in our profile (Additional file 2: Table S1). *KDM4B* and *XBP1* are part of the interaction network with *ESR1* while *SPDEF*, *FOXA1*, *GATA3*, and *MYB* are linked to *AR* (Fig. 5). Therefore, our data is consistent with and extends the information provided by prior studies. As the samples used in our study were from AH in patients without breast cancer, these genes define alterations in pathways that may contribute to the formation of premalignant breast lesions. The prominent role of estrogen signaling in the network is consistent with the success of anti-estrogen treatments in preventing progression of AH to carcinomas [55, 56].

Signaling networks in AH may also include mRNAs and proteins where levels are unchanged, but their activities are stimulated by interactions with other proteins causing post-translational modifications (e.g., by phosphorylation) and formation of larger complexes. Therefore, the 1st-order networks were also interrogated using KEGG pathways. This yielded a complex network of 306 genes and again identified over-representation of ERB-B signaling. WNT signaling was also found to be significantly over-represented in AH (Table 3). While SFRP1 can antagonize WNT signaling, it has been shown to also bind other proteins such as RANKL and thrombospondin, as well as affects signaling and responses via ERα, TGFβ, and p53 [30–32]. Furthermore, loss of *SFRP1* is an early event observed in the MCF10A progression series [57]. Therefore, we tested whether loss of *SFRP1* expression may regulate a portion of the genes within the AH signature. Inhibition of *SFRP1* in immortalized normal breast epithelial cells mirrored the changes in expression of 13 genes that were differentially expressed in AH (Fig. 6b). Re-expression of *SFRP1* in MCF7 cells reversed the changes in expression of 9 of the 13 genes tested (Fig. 6d). The presence of an SFRP-regulated gene network was also conserved in mouse mammary tissues (Fig. 6c). Consistent with these findings, the *Sfrp1*−/− mice exhibit precocious side-branching and hyperplasia of ductal/lobulo-alveolar units [35]. Together, these data demonstrate a role for SFRP1 in driving a portion of the signature found in AH.

Several of the SFRP1-regulated genes are involved in signal transduction. Loss of SFRP1 expression had consistent effects resulting in increased expression of *FOXA1*, *ERRB4*, and *KDM4B* in both TERT-siSFRP1 cells and in mouse mammary tissues. FOXA1 is a pioneer factor which can open up chromatin to allow for access to ERα transcriptional sites [58–60]. KDM4B is a histone demethylase which is upregulated in ERα+ breast tumors and can regulate the expression of both ERα and FOXA1 as well as modulate ERα and p53 signaling [61–64], ERBB4/HER4 is critical for progesterone receptor (PR) expression [65] and has also been suggested to be responsible for promoting an autocrine proliferation pathway induced by estrogen [66, 67]. The increased expression of *FoxA1*, *Kdm4b*, and *Erbb4* a mammary glands of *Sfrp1*−/− mice is consistent with the increased proportion of PR expressing cells and proliferation [32].

Analysis of ERα activity and endogenous ERα targets in our human cell lines also exhibited enhanced ERE reporter activity when SFRP1 was knocked down and repressed reporter activity when SFRP1 was re-expressed [32]. To confirm previous studies suggesting SFRP1 control of estrogen-induced PR expression, we added rSFRP1 to explants of normal breast tissue and demonstrated tempered induction of PR protein by estrogen (Fig. 7). Deletion of *Sfrp1* in mice also resulted in enhanced estrogen-stimulated responses and occasional hyperplasias, but was not sufficient for the development of spontaneous mammary tumors [32, 35]. Therefore, loss of *SFRP1* expression appears to be a key driver leading to broader alterations in gene expression and permitting increased signaling through ERα and derangements in the ERB-B and WNT pathways as well.

Although considered a “benign” lesion because progression to invasive cancer is relatively low, AH presage at least 40,000 breast cancer diagnoses annually [1, 68]. Therefore, intervention at this early stage offers the possibility of prevention of breast cancer. However, to minimize overtreatment, it is important to identify biomarkers discriminating the small subgroup of women with AH for whom risk is sufficiently high to warrant intervention. Molecular profiling of ductal carcinoma in situ aids in identifying women who may omit radiation following breast conserving surgery using a 12-gene panel (7 biomarkers, 5 reference genes) [69, 70]. None of these 7 biomarkers were detected among the 1039 genes that were differentially expressed in AH compared to histologically normal epithelium (Additional file 2: Table S1). While the 99-gene signature discriminates AH tissues (Fig. 3), the study is not able to access the utility of the signature in assigning risk. Larger cohorts of pure AH with follow-up of > 20 years for validation of predictive signatures are needed to identify women with AH who will benefit from interventions and reduce the potential for overtreatment [2].

## Conclusions

These results identify differentially expressed genes that can be used to assist AH diagnoses. Loss of *SFRP1* expression is a significant regulator of transcriptional profiles in AH and acts, in part, to limit estrogen signaling. These results support a broader role for SFRP1 in coordinating estrogen-induced responses and WNT signaling in normal breast epithelial cells.

## Additional files

Additional file 1: **Figure S1.** The relative expression levels of *SFRP1* mRNA are reduced in TERT-siSFRP1 cells and elevated in MCF7-SFRP1 cells. Total RNA was isolated from each cell line in triplicate for real-time PCR analysis. The level of *SFRP1* mRNA was normalized to the amplification of *ACTB* mRNA, which was performed in parallel wells for each cell line. Bars represent mean ± SEM *SFRP1/ACTB* and are expressed as relative expression of control cells (TERT-pSUPER or MCF7-pCDNA). ***p < 0.001 (significantly different from control cell lines using Student’s *t* test). (JPG 182 kb)

Additional file 2: **Table S1.** Probesets that are differentially expressed (1039 probesets). **Table S2.** Probesets selected by *p* < 0.005 used for hierarchical clustering by AGNES (99 genes). **Table S3.** Probesets selected by PAM (139 genes). **Table S4.** Zero-order gene network. **Table S5.** Primers for RT-qPCR. (XLSX 204 kb)

## Data Availability

The gene expression microarray data have been deposited in the Gene Expression Omnibus repository. The series record is GSE118432.

## Abbreviations

ADH: Atypical ductal hyperplasia
AH: Atypical hyperplasia
ALH: Atypical lobular hyperplasia
E2: 17β-estradiol
ERα: Estrogen receptor alpha
FEA: Flat epithelial atypia
FFPE: Formalin-fixed paraffin-embedded
HNB: Histologically normal benign
IHC: Immunohistochemistry
LCIS: Lobular carcinoma in situ
PR: Progesterone receptor
RT-qPCR: Reverse transcriptase quantitative polymerase chain reaction
