# Supplementary figures and images for: Gene expression signature of atypical breast hyperplasia and regulation by SFRP1
Source: Breast Cancer Res. 2019 Jun 27;21:76. doi: 10.1186/s13058-019-1157-5 (PMC6598287; doi:10.1186/s13058-019-1157-5)

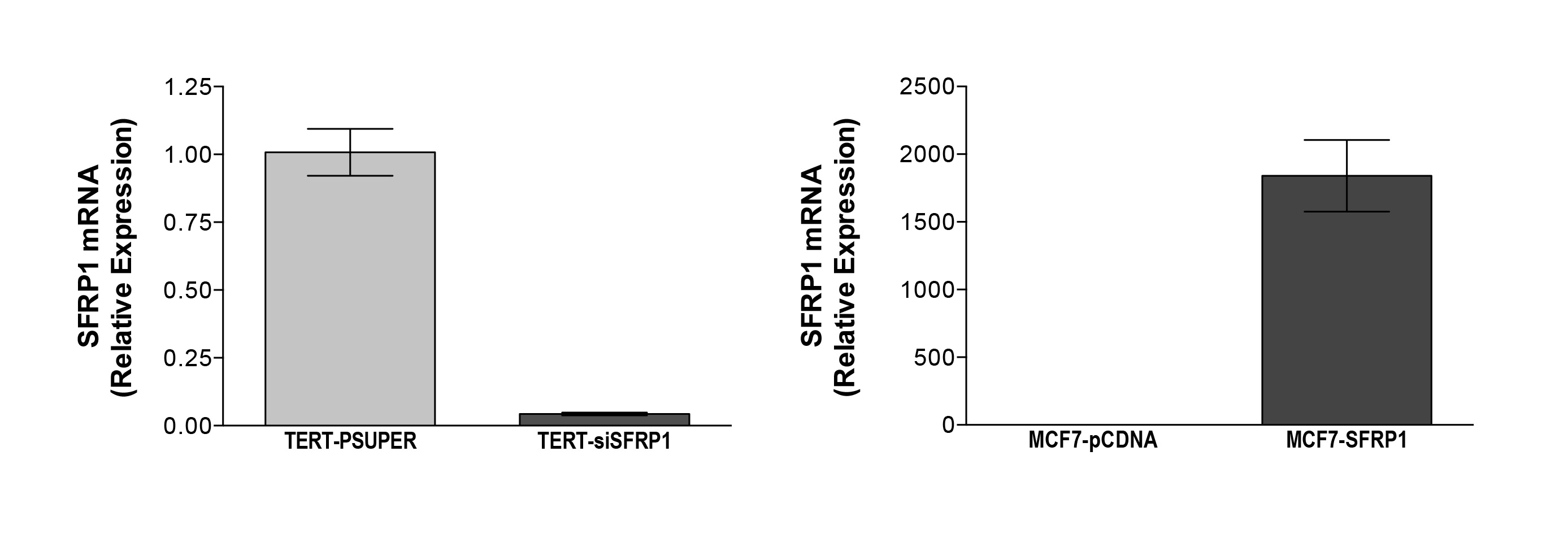

Supplement: Supplementary file 1 — Figure S1. The relative expression levels of SFRP1 mRNA are reduced in TERT-siSFRP1 cells and elevated in MCF7-SFRP1 cells. Total RNA was isolated from each cell line in triplicate for real-time PCR analysis. The level of SFRP1 mRNA was normalized to the amplification of ACTB mRNA, which was performed in parallel wells for each cell line. Bars represent mean ± SEM SFRP1/ACTB and are expressed as relative expression of control cells (TERT-pSUPER or MCF7-pCDNA). ***p < 0.001 (significantly different from control cell lines using Student’s t test). (JPG 182 kb) [file 13058_2019_1157_MOESM1_ESM.jpg]
